# Team Synergies in Sport: Theory and Measures

**DOI:** 10.3389/fpsyg.2016.01449

**Published:** 2016-09-21

**Authors:** Duarte Araújo, Keith Davids

**Affiliations:** ^1^CIPER, Spertlab, Faculdade de Motricidade Humana, Universidade de LisboaLisboa, Portugal; ^2^Centre for Sports Engineering Research, Sheffield Hallam UniversitySheffield, UK

**Keywords:** complex adaptive systems, synergies, team sport performance, ecological dynamics, group behavior, performance analysis

## Abstract

Individual players act as a coherent unit during team sports performance, forming a team synergy. A synergy is a collective property of a task-specific organization of individuals, such that the degrees of freedom of each individual in the system are coupled, enabling the degrees of freedom of different individuals to co-regulate each other. Here, we present an explanation for the emergence of such collective behaviors, indicating how these can be assessed and understood through the measurement of key system properties that exist, considering the contribution of each individual and beyond These include: to (i) dimensional compression, a process resulting in independent degree of freedom being coupled so that the synergy has fewer degrees of freedom than the set of components from which it arises; (ii) reciprocal compensation, if one element do not produce its function, other elements should display changes in their contributions so that task goals are still attained; (iii) interpersonal linkages, the specific contribution of each element to a group task; and (iv), degeneracy, structurally different components performing a similar, but not necessarily identical, function with respect to context. A primary goal of our analysis is to highlight the principles and tools required to understand coherent and dynamic team behaviors, as well as the performance conditions that make such team synergies possible, through perceptual attunement to shared affordances in individual performers. A key conclusion is that teams can be trained to perceive how to use and share specific affordances, explaining how individual’s behaviors self-organize into a group synergy. Ecological dynamics explanations of team behaviors can transit beyond mere ratification of sport performance, providing a comprehensive conceptual framework to guide the implementation of diagnostic measures by sport scientists, sport psychologists and performance analysts. Complex adaptive systems, synergies, group behaviors, team sport performance, ecological dynamics, performance analysis.

## Introduction

Sport is a human activity characterized by particular organization and functioning in given performance contexts. The ecology of sport is not only distinguished by physical characteristics of locations at which player activity takes place, but also by its social significance and cultural aspects. A factor of interest for audiences as well as sport scientists and performance analysts, is to observe which players or teams succeed in competition, characterized by a complex interaction of physical (e.g., surfaces, areas, weather conditions) and social (e.g., rules) constraints specific to each sport. In team sports, the overarching aim of a team is to score more points/goals than the other team, implying an initiative to score (attacking), and to prevent the other team from scoring (defending). This distinction between attacking and defending is somewhat of an oversimplification, because there are sports where a team can be defending a lead, when in possession of the ball, and a losing team without the ball may be pressurizing space or the opposition ball carrier to re-gain possession. It may be more appropriate to talk about each team’s offensive and defensive capacity (Mateus, 2005). In each case, a specific feature of team ball sports is the flow of interactions between cooperating and competing players, with or without the ball, to achieve the competitive performance aims of each team (Mateus, 2005). The aims of the teams are thus mutually exclusive during competitive performance. Essentially, each team tries to prevent the implementation of the performance strategy of the other team, as well as dissipate their tactical actions (Davids et al., 2005). The result is the emergence of a complex web of interactive behaviors of players and teams during competition.

Team sport competitions have been conceptualized as complex dynamical systems composed of many interacting parts (e.g., Davids et al., 2005). A dynamical systems approach to sport performance describes how patterns of coordinated movement come about (‘emerge’), persist, and change. It builds on the insight that teams, as social systems, consist of a large number of interacting parts, endowing them with a capacity for spontaneous pattern formation or self-organization. The spontaneous creation of coherent macroscopic patterns (e.g., team coordination) is important scientifically, and the resulting macroscopic patterns of the dynamics of one or a few collective variables or order parameters can be studied carefully (e.g., the cluster phase; Duarte et al., 2013), without needing to record all the microscopic states of the individual parts (e.g., the movement of each player; see Kelso, 1995). Conversely, when the dynamics of macroscopic phenomena have been identified, the contributions of relevant dynamical components (e.g., the movement of certain players) to the overall dynamics may be investigated in a top–down fashion (Araújo et al., 2015).

Performers can generate behavioral patterns that are tightly coordinated with environmental events (e.g., the match configuration), in order to achieve a specific performance goal. In team sports, athletes are surrounded by physical (e.g., gravity, altitude, ambient temperature) and social (e.g., audience, rules of the game, local rivalries) constraints. Successful performance in sport is predicated on the constraints of an individual’s perceptual and action capabilities, and is grounded on information used for action selection and goal achievement (Araújo et al., 2006).

Assuming the mutuality and reciprocity between the performer and the environment, i.e., the performer-environment system as the key level of analysis, implies, not only an active agent, but also that the environment is an active part of a system, facilitating specific behavioral outcomes (Davids and Araújo, 2010). One consequence of this account is that behavior can be understood as self-organized, in contrast to organization being imposed from inside (e.g., by instructions of the team captain in sports teams) or outside (e.g., directions of the coach). Team performance does not always need to be prescribed by internal or external structures, due to inherent self-organization tendencies that exist for exploitation in sports teams (Araújo et al., 2016) yet traditional sports psychology and pedagogical practice has decreed that internal/external prescription is the default mode to face this challenge (e.g., Lidor and Henschen, 2003). Ecological dynamics suggests that, from a player’s point of view, the task is to exploit physical (e.g., pitch characteristics determined by federation rules) and informational (e.g., perception of the movements of other players) constraints to stabilize emergence of intended behaviors. Constraints have the effect of reducing the number of configurations available to a dynamical sports team (conceptualized as a social adaptive system) at any instance. In team sports competitions, coordination patterns (individual or collective) emerge under constraints as less functional states of organization are dissipated. Every team sport presents its own set of interacting constraints that helps define competitive functioning. This view contrasts with the traditional use of team statistics and notations that are often used to mechanistically operationalize team sports performance in a data-driven way (e.g., frequency counts and averages in sports: see Vilar et al., 2012; Travassos et al., 2013; Couceiro et al., 2016).

In fact, the ever-increasing successful implementation of new technologies in sports inevitably leads to sequential stages of: data capture, pre-processing, transfer, post-processing, visualization, and analysis. This relentless emphasis in current performance analysis (PA) methodologies involves a high volume, variety and complexity of data, to meet the demands of practice, evaluation and training in different sports.

As fundamental theories have failed to make any reliable analysis or predictions around football, most contemporary research has focused on probabilistic models (e.g., Owramipur et al., 2013). Nevertheless, and despite the success of some of these methods, they rely only on the overall information retrieved from previous matches, without considering on-the-fly information retrieved during a current match, such as each athlete’s positional and physiological data, nor producing any macroscopic tactical metric inherent to such data (see Couceiro et al., 2016) for a critical analysis of these methods. A critical point here is that, typically, notational analysis tends to *describe* performance, but omits reference to the *whys* and *hows* that underlie the structure of many recorded behaviors, which would clearly define their functional utility (McGarry, 2009). Recently, Gudmundsson and Horton (2016), in an exhaustive review of measures of team behavior in “invasion sports” (i.e., association football, hockey, rugby union), proposed how information obtained from spatio-temporal data (Araújo et al., 2004; McGarry et al., 2014) could be categorized in what they called a “coherent framework.” The categories of this framework were: (i) description of variables that can be obtained from spatio-temporal data (i.e., player trajectories, sequence of events); (ii) divisions of the playing area; (iii) players’ interactions and temporal sequences of events (captured by networks and related metrics); (iv) types of information obtained by data mining techniques (labeling events, predicting future events, identifying formations, identifying plays and group movements, segmenting a match); (v) metrics to measure player and team performance; and finally (vi) visualization techniques used to present metrics calculated from the visuo-spatial data. This framework, as well as others (Bartlett et al., 2012), is useful, but its rationale is essentially methodological, focusing on the production of an operational account of performance. This means that it has limited use for understanding team behaviors, only a capacity to measure them and operationally define them. The questions that arise are: what is the meaning of these metrics? What theoretical concepts of a theory of team behaviors can be used to explain how these behaviors emerge?

To address these, and related, questions PA should not focus on production of operational measures and performance descriptors only. Rather, PA could really benefit from the development of a detailed account providing theoretical support for interpretation of data that arise from the descriptive measures. Ecological dynamics is such a theoretical framework for studying behaviors in team games (Davids et al., 2005; Araújo et al., 2006). It has the advantage of recognizing the ‘flexibility’ of social systems (i.e., teams), and its principles can explain how the same performance outcomes can emerge from different behavioral patterns. More than a decade ago (e.g., Araújo et al., 2004; Davids et al., 2006), we began to develop a theory of interpersonal behaviors in team sports, applying concepts such as that of “superorganism” to characterizing a team (Araújo, 2006; Duarte et al., 2013; Araújo et al., 2016). This path led us to the “team synergy” hypothesis and its operationalization, presented here (see Passos et al., in press for an extensive synthesis of findings and methods applied in different team sports). The hypothesis of team behavior as a synergy is possible only if we conceive the origin of a team synergy as an emergent phenomenon, which is a consequence of players’ perceptual attunement to shared affordances, as framed by the ecological dynamics approach that we briefly overview next. But before that, we concisely present an overview of other competing hypotheses exiting in the sport sciences literature.

## Some Alternative Hypotheses to Explain Team Behaviors

There are a limited number of hypotheses in the literature to explain team behaviors (see Araújo and Bourbousson, 2016, for a review). Here we outline the two more prominent proposals for the team synergy hypothesis: the hypothesis of shared knowledge grounded in the socio-cognitive approach and the participatory sense-making hypothesis grounded in the enactive approach.

The assumption of shared knowledge is predicated on the possession by team members of mental models that provide a basic shared understanding of how to achieve desired performance outcomes (Ward and Eccles, 2006). It is argued that team efficacy could increase when a sophisticated, global and comprehensive mental representation of a performance context of a collective action is somehow shared by all players and put into practice. Lack of coordination between the intentions of individual performers and those of the team imply that a shared cognitive state has yet to be achieved, with resulting difficulties in team performance (Eccles, 2010). In this view, it is the construction and updating of the individual’s mental model that explains how multiple performers may simultaneously act together. As many team members are simultaneously coordinating, the amount of similarity within individuals’ representation acting together becomes a key feature, thus indicating a state of shared understanding within the team. However, knowing “*who knows what*” at each moment of a match would involve an immense computational load for a representational system. Particularly, the mechanism to explain re-formulations of a team member’s schema, when changes occur in the content of another member’s schema, has proved difficult to verify (Mohammed et al., 2000). A key difficulty is to justify how mental representations exist beyond the mind of an individual organism and can be somehow shared in a collective representation (Riley et al., 2011; Silva et al., 2013).

Another alternative is the participatory sense-making hypothesis which suggests that team coordination processes should be investigated by reconstructing how individual ‘cognitions’ articulate during performance environments. With the claim that a performer possesses a unique “interiority,” this hypothesis has given a special attention to the implicit ways how each performer experiences his/her ongoing activity. Participatory sense-making processes refer to how the meanings that each performer internally builds from his/her activity corroborate with the meanings simultaneously built by co-performers, and how this participation in sense-making is experienced (De Jaegher and Di Paolo, 2007; Froese and Di Paolo, 2011). From this perspective, it is assumed that putative ‘participatory sense-making’ emerges from a cooperative effort (De Jaegher and Di Paolo, 2007). Performers working together achieve the experience of mutual engagement in real-time. Any divergence in how each member experiences a performance situation leads to varied degrees of participation in sense-making, variations in feelings of connectedness with the other, and variations in expectations for actions from others (e.g., Bourbousson et al., 2010a, 2015). This enactive approach tries to avoid representationalism, but by being grounded in the “interiority” of each individual it needs to operationally define what the internal sense-making process is and contrast it with representations of lived situations which are heavily reliant on memory. In fact, it should be clarified why there is a need to add the label “sense-making” to the process of team coordination, unless we are operating in an approach that overstates the asymmetry of organism and environment (Fultot et al., 2016). The idea proposed by enactive theorists that meaning (about the world) is internally built is not operationally defined in terms that exclude representations (prompting the question: where is meaning constructed, and how, and in what form?) and therefore, may be subject to the same criticisms as the shared knowledge hypothesis about the overreliance on mental representations, individual or collective.

In general, both the participatory sense-making and the shared knowledge hypotheses rely on data from *a posteriori* verbalizations of team sports performers. Therefore, the organization of behavior, from these perspectives, is mainly understood and derived from the verbalized conceptions and perceptions about behavior, not from the actual behaviors themselves. From these perspectives, overt behavior and its organization in contexts like team sports is a surrogate of verbalized shared sense-making or knowledge, without a self-organization of its own. In contrast to these perspectives, it is argued here that behavior has an organization that goes beyond what a performer possesses (verbal information expressing knowledge or its interiority). Rather it is deeply rooted in the specific circumstances of behavior, in which continuous performer-environment interactions are not determined by one component of such a system.

## Ecological Dynamics and Team Sports Performance: The Shared Affordances Hypothesis and its Relationship with Team Synergies

Understanding group coordination from the perspective of ecological dynamics requires investigating the dynamical principles that underpin the self-organizing patterns of group dynamics in performance contexts (Araújo et al., 2016; Passos et al., in press). Interpersonal patterns at the behavioral scale can be understood and modeled in terms of their own dynamics, without the need to investigate each performer’s particular movement patterns (nor their verbalizations of what these might be; Schmidt et al., 2011). Analyses of group dynamics capture the continuous interactions of system components to form stable behavioral patterns, characterized as attractor states of system dynamics (Schmidt et al., 1990). Important for the study of group dynamics is the fact that synchronization processes (i.e., temporal coordination of unfolding events in a system) have been found to occur between organisms that are connected, not only mechanically, but also informationally. In this approach, information is conceptualized in the specific patterns in surrounding energy distributions (e.g., light, sound, neural) that can specify properties of the world, meaning that the mapping from patterned energy distributions to properties of the world is direct (not reflected upon or inferred from representations of these relations). Much research has demonstrated how this dynamical synchronization process can operate predicated on information to produce coordinated timing of interpersonal interactions (e.g., Richardson et al., 2007). In these studies, the only possible way that the rhythmic units could have interacted was via the information available to the visual systems of the human participants. Importantly, distributions of energy surrounding an organism, when properly described, are rich with information that specify action-relevant properties of the world. More to the point, specificational information is the invariant structure of (low) energy distributions lawfully structured by emergent individual–environment interactions that, because of that lawfulness, specify relationships of each individual with the environment. This is precisely the type of invariant structure that supports inter-player coordination in sports teams.

Understanding of the informational basis for the emergence of group coordination, and the dynamics that permit the self-organization of coordinated group action to occur is, therefore, a priority for research from this viewpoint. This is not because it implies athletes do not have intentions, thoughts and mental states, but rather because research demonstrates how integrally intertwined are body (e.g., nervous, physiological, psychological, emotional) and contextual (e.g., social, cultural, climate, altitude) sub-systems, during interpersonal coordination (Marsh et al., 2009). Adopting an environment-individual system perspective (Richardson et al., 2008; Järvilehto, 2009; Marsh et al., 2009), an ecological approach proposes that knowledge of the world is based upon recurrent processes of perception and action through which humans perceive affordances (i.e., opportunities for action, see Gibson, 1979). The concept of affordances presupposes that the environment is directly perceived in terms of what actions a performer can achieve within a performance environment (i.e., it is not dependent on a perceiver’s expectations, Richardson et al., 2008). Moreover, to perceive an affordance is to perceive how one could act with respect to an environmental layout. This way, affordances are neither external properties of an environment nor are they representational properties of mind; they are relational properties of a performer-environment system, which capture the link between individual and environment and are perception-action system specific (Fajen et al., 2009). Affordances capture the action specific relations that exist between the skills/capacities of an individual performer and the action relevant properties of a task, including social tasks (Heft, 1989; Withagen et al., 2012). The theory of affordances is based on the dual interdependence of perception and action, where affordances are the primary objects of perception, and action is the realization of affordances (Araújo et al., 2013). Consequently, it is the role of scientists to discover information in ambient energy arrays that specifies action-relevant properties (such as team synergies) and to show how team synergies may be constrained by such information (Araújo et al., 2006).

Recently, Silva et al. (2013) challenged the hypotheses for team coordination described in the previous section. We argued that these hypotheses were predicated on ‘knowledge about’ the environment, which can be used to share knowledge and participatory sense-making and consequently organize action. Rather, during competitive performance, the organization of action by perceiving surrounding informational constraints is expressed in ‘knowledge of’ the environment. This crucial distinction emphasizes that the perception of shared affordances (for others and of others) underpins the main communication channel between team members during team coordination tasks. We grounded these explanations on Reed’s (1996) conception of affordances where affordances are resources in the environment. In this view, the relative persistence of some affordances in the animal’s environment has given rise to the evolution of several distinct action systems, enabling the animal to take advantage of these affordances (Reed, 1982, 1996). This view suggests that evolution gives rise to animals that are capable of taking advantage of the relatively persistent affordances in a performance environment. These resources in a performance environment have imposed selection pressures on some group of individuals, causing them to evolve perceptual systems to perceive these relations. Predicated on the key ideas of Reed, we went as far as arguing that affordances are collective environmental resources that have existed prior to the upskilling of the individuals that came to perceive and use them. At the timescale of team sports performance, these ideas can be taken to imply that more successful teams are composed of athletes who have learned to perceive shared affordances for and of other players.

Reed (1982, 1996) also asserted that the exertion of selection pressures by affordances is a dominant force in evolution by natural selection. However, an important criticism of this view is that the environment not only shapes individuals, but individuals also modify their environment (see Withagen and Van Wermeskerken, 2010). More precisely, individuals and their environments evolve together. In fact, Heft (2007) brought the process of niche construction to attention when arguing that the human environment is largely a product of social processes, aligned with an important discussion about the process of “niche construction” in evolutionary psychology (Odling-Smee et al., 2003). The phrase “niche construction” refers to how the modifications that the organism brings about in its environment can create new evolutionary equilibria and trajectories (Withagen and Van Wermeskerken, 2010).

Aligned with the ideas of Reed, we concur that affordances constitute the context of selection, but updating our hypothesis of shared affordances we take the dynamic view presented by Withagen and Van Wermeskerken (2010) that individuals’ dissolution and construction of affordances change this context, demonstrating the key roles affordances play in learning, development, and evolution. This clearly signifies that individuals do not passively evolve so as to fit in a preexisting environment. Rather, individuals and their environments actively co-evolve, and each individual’s modification of the environment has a constitutive role in this co-evolution process. Individuals modify their (social, physical) environment, which can change the selection pressures to which they are exposed. Lewontin (1983) emphasized that there are constraints on the co-evolution of animal and environment. Hence, contrasting with the ideas of Reed, Lewontin would not conceive the physical properties of the world as a resource that exerts selection pressures, but would conceive of them as constraints on the evolution of the individual–environment system. This ideation indicates, that affordances arise along with the evolution of action systems and provide the context of selection and adaptation after they have evolved (Withagen and Van Wermeskerken, 2010). In short, niche construction not only requires the utilization of affordances, it also consists of a change in the affordance layout. Hence, individuals often create and dissolve affordances, with other individuals (members of a species or, in humans, sports teams) being exposed to these modified environments as new members of a group (Withagen and Van Wermeskerken, 2010). Thus, the ecological inheritance from one group to the next encompasses an inheritance of affordances. It is important to emphasize, however, that changes in the affordance layout are not exclusively the result of individual activity. Indeed, as mentioned above, geo-physical (including built environments) and social processes can also alter the affordances in an individuals’ eco-niche, such as a training setting. Furthermore, it is important to reiterate that creating a niche is of course not always evolutionarily consequential – it does not have to change the context of selection. However, as we have discussed, niche construction can alter the developmental trajectory of a collective system in small or extensive ways, which could be significant or not for group performance.

Extending this idea to the level of interpersonal interactions, ecological dynamics predicts that the presence of others extends action possibilities that are realizable by individuals to action possibilities realizable by groups. Indeed, Gibson (1979) argued that ‘behavior affords behavior’ (p. 135) and that it was important to note that the ‘richest and most elaborate affordances’ (p. 135) of all are affordances of other people in social contexts. What do these rich insights imply for understanding of coordination in team sports? The suggestion is that affordances can be perceived by a group of individuals trained to become perceptually attuned to them (Silva et al., 2013). Given the mutually exclusive performance aims of both teams in a sports contest, affordances are shared and sustained by common task goals of team members who cooperate and compete to achieve group success. From this perspective, team coordination depends on the collective attunement of individual athletes in a team to shared affordances (Silva et al., 2013). Through practice, players can become perceptually attuned to affordances *of* others and affordances *for* others during competitive performance, and can refine their actions (Fajen et al., 2009) by adjusting behaviors to functionally adapt to those of other teammates and opponents. Moreover, individuals in a team can act in a way to create competitive circumstances (the affordances) that are favorable to them. For example, by pressuring opponents to play more in one zone of the playing area, this strategy can create affordances for attacking play by freeing other areas of the field/court/pitch. This active construction of affordances can be trained too. These processes enable a group of players to act synergistically with respect to specific match circumstances (Araújo et al., 2015). Importantly, shared affordances are predicated on perception, whereas synergies regard action, meaning that a synergy is a physical process that realizes a task goal under the guidance of affordance-specific information.

### Synergies and Ecologies

A synergy is a functional concept, not a structural, component-based concept. In analyses of human movement it relates directly to explanations of coordination processes in multi-articular systems such as the body of an athlete or teams that compete in sport. Turvey (2007) defined a synergy as a group “of relatively independent degrees of freedom that behave as a single functional unit – meaning that the internal degrees of freedom take care of themselves, adjusting to their mutual fluctuations and to the fluctuations of the external force field, and do so in a way that preserves the functional integrity of the” group (p. 659). More broadly a synergy is “[T]he adaptive fit of parts of a system to each other and to the system as a whole” (Turvey and Fonseca, 2014, p. 152). Considering this definition, in a collective system, a synergy is a task-specific organization of individuals, with the degrees of freedom of each individual having the potential for coupling, enabling the degrees of freedom of different individuals to regulate each other (Bernstein, 1967). Synergies require the modulation of fewer parameters than the separate control of each degree of freedom, in order to bring about coordinated movement. This system capacity reduces the need for control of each degree of freedom, and allows for compensatory variability in one element of the synergy by another. Importantly, coordination processes that characterize a synergy are not predicated on a cooperativity of individual structural components, but rather on the cooperativity of their functional roles. In other words, synergies being task specific, they are not conceived by design; they are not hard-wired to behave in a pre-arranged way, and therefore the context-dependent functionality of synergy components should always be recognized (Turvey, 2007). Synergies are, thus, *context-dependent, time-evolving dynamical systems* (Thelen and Smith, 1994) that, according to circumstances, self-organize several, individual system components in an appropriate and timely fashion.

An important feature of a team synergy is the capacity of one individual (e.g., a player in a team) to influence behaviors of others (Riley et al., 2011; Araújo et al., 2016). Decisions and actions of players forming a synergy should not be viewed as independent, explaining how multiple players synchronize activities in accordance with dynamic performance environments in fractions of a second (Silva et al., 2016). The coupling of players, as independent degrees of freedom, into synergies is based upon perception-action systems in a social context supported by the collective perception of shared affordances (Silva et al., 2013). As we elucidate in the next section, research has demonstrated that inherent degeneracy (i.e., flexibility) in perception-action systems provides the neurobiological basis for the diversity of actions required to negotiate information-rich, dynamic social environments toward a task goal, as well as providing a huge evolutionary fitness advantage (see Seifert et al., 2016). Therefore, the relationship between the characteristics of the environment and each individual’s skills and capacities can be captured in the perception of an affordance (Withagen et al., 2012). System degeneracy between individuals would reflect each individual’s actualization of an affordance through various coordination patterns. That is, the same affordance can be actualized with different coordination patterns as individuals interact with task and environmental constraints.

In line with this notion, an ecological dynamics perspective seeks to predict conditions under which individuals are better able to coordinate movements with others, and which features of a situation facilitate/perturb interpersonal coordination in completing some task. This view has major implications for designing experiments for studying team performance behaviors, as well as for practice and training design. Brunswik (1956) was likely the first psychologist advocating theoretical principles for sampling the features of a task, using the concept of ‘representative experimental design.’ He argued that perceptual variables incorporated into experimental designs should be sampled from the performer’s typical performance environment, to which behavior is intended to be generalized. This powerful idea implies that experimental designs, aligned with an ecological dynamics perspective, need to focus on player-player-environment interactions that can be elucidated in compound variables specifying functional collective behaviors of teams (e.g., a given attack configuration), underpinned by interpersonal synergies created between performers (see Araújo et al., 2015, for a review). An important pedagogical principle in sport is the need to ensure that there is adequate ‘sampling’ of informational variables from the performance environment in a practice task, ensuring that modified training tasks will correspond to an actual competition context so that important sources of information are present (Araújo and Davids, 2015).

Ecological dynamics analyses of team sports have attempted to explain how interactions between players and information from the performance environment constrain the emergence of patterns of stability, variability and transitions in organizational states of such team synergies. With motion sensors, it is possible to examine interpersonal rhythmic coordination of movement (Kelso, 1995). The emergent coordination patterns in team sports are channeled by surrounding constraints that structure the state space of all possible configurations available to the team game as a complex system (Davids et al., 2006). For example, the surrounding patterned energy distributions (i.e., information) that performers can perceive act as important sources of information to support their decisions and actions (e.g., reflected light from the ball; Araújo and Davids, 2009).

By using tracked positional data, recent studies have started to reveal how players and teams continuously interact during competition. For example, it has been observed that teams tend to be tightly synchronized in their lateral and longitudinal movements (Vilar et al., 2013), with a counterphase relation in their expansion and contraction movement patterns (Yue et al., 2008), commonly instigated by changes in ball possession (Bourbousson et al., 2010b). The coordination patterns observed showed compensatory behaviors within the team, an essential characteristic of a synergy (Riley et al., 2011). Under this theoretical rationale, several existing variables obtained from spatio-temporal data can be organized according to the team synergy hypothesis, and synergy properties can also guide the discovery of new variables.

## Properties of Team Synergies and their Measurement

There are slightly different perspectives on the relevant properties of a synergy (Turvey, 2007; Latash, 2008; Araújo et al., 2016). Latash (2008) identified characteristics that should be met for a group of components to be considered a synergy, including: (i) sharing patterns, where the components should all contribute to performance of a particular task; (ii) error compensation, where some components show changes in their contributions to a task, compensating for a component that may not be making a relevant contribution; and (iii), task-dependence, the capacity of a synergy to change its functioning in a task-specific way, or to form a different synergy for a different purpose based on the same set of components. Task dependence is an expression of redundancy (Bernstein, 1967), or more generally degeneracy (Davids et al., 2006; Seifert et al., 2016), defined as the capacity of structurally different components to perform a similar, but not necessarily identical, function with respect to context (Edelman and Gally, 2001). Alternatively, Riley et al. (2011) identified two properties of a synergy: (i) dimensional compression, where the degrees of freedom that potentially are independent become coupled so that the synergy has fewer degrees of freedom (a lower dimensionality) than the set of components from which it arises (Bingham, 1988). Interestingly, the behavior that emerges from the interactions among degrees of freedom, not among the structural components, constitutes a second level of dimensional compression. Dimensional compression at both stages results from imposing environmental, task and individual constraints, which couple components so they change together, rather than independently. The second property of a synergy for Riley et al. (2011) is reciprocal compensation and it is a less biased description of what Latash (2008) described as “error compensation.” To label a behavior as “error” is to assume normativity in observers. Another possible label for an error is creativity, even though creativity goes much beyond reciprocal compensation (Hristovski et al., 2011).

Therefore, we address four properties of a synergy which are: (1) dimensional compression (Bingham, 1988; Riley et al., 2011), (2) reciprocal compensation (Latash, 2008; Riley et al., 2011); (3) Interpersonal linkages. According to Latash (2008), who termed this property “sharing patterns,” a way to quantify the amount of sharing is the matching of the sum of the individual contributions to the task, where the overall measurement of task performance may be related to the dimensional compression property. In contrast, and inspired by Ingold (2015), we address this property in relation to the individuality of each element of a synergy, and therefore, the different ways they can link together; and (4) degeneracy (Davids et al., 2006; Latash, 2008; Seifert et al., 2016). However, degeneracy may be seen as a more general property that can be expressed in different ways, compared to the previous properties which are more specific. This broader view is needed because it shows adaptability across tasks (e.g., competitive matches) and across changes in system components (e.g., players) as occurs in a sport team. Next we describe how these properties can be measured in team sport performance.

### Dimensional Compression: “One for All, All for One”

A synergy, conceptualized as a controllable organization of many individual degrees of freedom, is an organization of afference (perception). From an ecological dynamics point of view, shared affordances guide team behaviors. This implies that the afferent and efferent flows comprising a synergy unfold in a collective variable (or order parameter) – a measurable quantity that expresses a coherent relation among the synergy’s parts and processes (Turvey, 2007). The time-evolution of a synergy, subject to continuous afferent and efferent influences, can then be captured, in principle, by a single first-order equation in the order parameter.

#### Order Parameters

In a synergy, all the composing individual components become arbitrarily quick so that they can adapt instantaneously to changes in the control parameters (i.e., the variables that change the state of a system, Kelso, 1995). The synergy dynamics thus amount to those of the order parameters (collective variables), implying that the ordered states can always be described by very few variables, if in the neighborhood of behavioral transitions. In other words, the state (i.e., the synergy) of the originally high-dimensional system (i.e., the team) can be summarized by a few variables or even a single collective variable, the order parameter (Beek and Daffertshofer, 2014).

Self-organization in dynamical systems is predicated on dimensional compression. The work of Kelso (1995) exemplified a system order parameter in the form of ‘relative phase,’ in the study of human rhythmic movement coordination. Phase is an angular measure of an oscillator’s position within its cycle of movement, and relative phase is simply the difference in phase relations between two oscillators, This example of an order parameter captures the low-dimensional behaviors (the synergies) that arise from a high-dimensional system (e.g., sports teams). Relative phase describes the spatiotemporal pattern of rhythmic coordination and the changes in coordination that occur as sudden adaptations to manipulations of a system control parameter (e.g., movement velocity). In this case, the dynamics of relative phase is understood to reflect the behavior of a synergy (Kelso, 1995; Turvey and Carello, 1996).

In an attempt to capture group synchrony tendencies in a single variable (i.e., to capture dimensional compression, one of the properties of a synergy), Duarte et al. (2013) shed light on how the players composing a team influenced each other to create a collective movement at the team level. For this, instead of a relative phase value that only linked two players, Duarte et al. (2013) applied the cluster phase method (Frank and Richardson, 2010), based on the Kuramoto order parameter, to analyze the movements of 11 football players from two teams during a competitive football match to assess whole team and player-team synchrony. Synergistic relations within a whole team showed superior mean values and high levels of stability in a longitudinal direction compared to a lateral direction on field. Player-team synchrony revealed a tendency for a near in-phase mode of coordination. Also, the coupling of the two competing team’s measures showed that synchronization increased between both teams over time. This was likely the first time a formal measure of dimensional compression was used to characterize team synergies in sport and changes to them throughout the course of a competitive match between two professional teams. In sport sciences, there are team-based variables that may be precursors of measures for dimensional compression. It is important to clarify that these spatial team-based variables are not measures of compression along any dimensional axis, nor measures of coupled degrees of freedom of elements that potentially could be independent. This is why they may be seen as preliminary attempts to measure dimensional compression of a team synergy.

#### Team-Based Variables

Coaches often discuss the importance of the “center of gravity” of a team (Gréhaigne et al., 2011). An operational approach to this tactical concept is a team’s center (also denominated centroid, or geometrical center). It has been used in various ways to evaluate intra- and inter-team coordination in team sports (e.g., Bourbousson et al., 2010b; Frencken et al., 2011; Travassos et al., 2012). Team centers can represent, in a single variable, the relative positioning of both teams in forward-backward and side-to-side movement displacements.

According to coaching knowledge, the team in possession of the ball possession should seek to create space by stretching and expanding on field (increasing distances between players), while a defending team should close down space by contracting and reducing distances between players. Such collective movements may be captured by specific measures of team coordination that quantify the overall spatial dispersion of players on field. The stretch index (or radius), the team spread and the effective playing space (or surface area) are quantities that have been used to assess such spatial distributions (e.g., Folgado et al., 2012; Moura et al., 2012; Silva et al., 2014a). Also, it is important for a successful team to outnumber the opposition (creation of numerical overloads) during different performance phases (attack and defense) in spatial regions adjacent to the ball, expressed through inter-team coordination. Inter-team coordination was recently examined through analysis of the distances separating the horizontal and vertical opposing line-forces in competing football teams (Silva et al., 2014b). This measure captures the existence of possible differences in players’ interactive behaviors at specific locations on field (e.g., wings and midfield sectors).

Individual playing areas of each player in a team can be delimited by Voronoi cells, offering a time-evolving analysis of the trajectories of these areas (Fonseca et al., 2013). A Voronoi cell contains all the spatial points nearer to the player to whom a cell is allocated, than to other players. By measuring the total area of all Voronoi cells in each team, one can obtain a graphic capturing the dominant ratio of one team over another (Fonseca et al., 2012).

In sum, although dimensional compression implies the formalization of a low-dimensional variable that captures the behavior of a synergy, several team-based variables exist that offer relevant heuristics to better explore new order parameters in future research.

### Reciprocal Compensation: “We will Cover Your Back”

Reciprocal compensation indicates that, if one player contributes more or less in his/her expected role, other team elements should adjust their contributions, so that task performance goals are still attained (Latash, 2008).

In studies of team synergies, the property of reciprocal compensation has received less attention from researchers, although Riley et al. (2011) have suggested the significance of the uncontrolled manifold approach (Scholz and Schöner, 1999; Latash et al., 2002), which assumes that coordinated movement is achieved by stabilizing the value of a coordination variable (e.g., cluster phase). In doing so, a subspace (i.e., manifold) is created within a state space of task-relevant elements (the degrees of freedom that participate in the task), such that within the subspace, the uncontrolled manifold, the value of the coordination variable remains constant (Riley et al., 2011).

Recently, we created the variable readjustment delay (Silva et al., 2016), that measured the delay in co-positioning by footballers in adapting to teammate movements. It is a measure of the coherence and fluency in teamwork, capturing team readiness and synchronization speed during attacking and defending team actions. Lower delay values indicate rapid readjustment movements and faster spatial temporal synchrony between players, whereas a larger readjustment delay might impede spatial-temporal synchrony of player movements. We sought to understand whether practice could influence changes in these measures of teamwork. Silva et al. (2016) found that players’ readjustment delay values decreased over the 15-week program in the study, evidencing faster readjustments of coupled players, showing how this synergistic property evolved in a team as a function of practice.

More research is needed on this property, both by exploring the relevance of existing variables, and by developing new variables more specific to the constraints of different sports.

### Interpersonal Linkages: “It Makes a Difference if the Pope is on Your Side”

Interpersonal linkages, also known as sharing patterns (Latash, 2008), or division of labor (Duarte et al., 2012; Araújo et al., 2015), refers to the specific contribution of each element to a group task (Latash, 2008). The behavior of each individual in a team is constrained by several factors like his/her position on the playing area (in relation to other teammates and opponents), strategic and tactical missions, playing phases (i.e., attacking and defending), game rules, etc. According to Latash (2008), sharing is equated to the sum of the individual contributions to the task. However, we term this property “interpersonal linkages” within teams, because when players work together, they do not lose their individuality to a momentary sum, they create properties at the team level and at the same time they establish links that persist. Based on the conceptualization of Ingold (2015), we briefly explain this idea.

This property of interpersonal linkages highlights the need to consider that each element is unique, and this implies an understanding about team behavior that is different from considering a team as a superorganism. For example, in social biology, a ‘colony’ of conspecifics may be regarded either as an aggregate of discrete organisms or as a single superorganism. In a recent essay, Ingold (2015) clarified that an aggregate of individuals joined by mutual self-interest, is different from a superorganism situated above the individuals, where individuals are fused together in a new entity. Forming a group by aggregation or by fusion imply different links among elements (Ingold, 2015). In aggregation, individuals meet along their surfaces, turning every such surface into an interface separating the contents on either side. In fusion, these surfaces partially dissolve, so as to yield a whole that is more than the sum of its parts. However, the portion that an individual might share with others is instantly ceded to this higher-level, emergent entity, and what is left to the individual remains exclusive to its owner. The whole may encompass and transcend its parts, but the parts have nothing of the whole (Ingold, 2015).

However, a sport team can go beyond assemblage and fusion. What happens in expert teams is simultaneously a new entity with properties beyond the individuals, and at the same time there are individuals who can contribute with their unique skills. Ingold (2015) calls this way of linking individuals “correspondence.” Correspondence, implies regarding individuals, not as closed-in entities that can be enumerated and added up, but as open-ended processes that *carry on*. For Ingold (2015), “carrying on” means that individuals wrap around one another. A sport team is not simply an articulation of independent components, nor a totality that ignores the unique skills of individuals; but ever-extending lives, and its synergies “reside in the way each strand, as it issues forth, coils around the others as is coiled in its turn, in a counter-valence of equal and opposite twists which hold it together and prevent it from unraveling” (Ingold, 2015, p. 11). A team is neither additive nor exponential, it is contrapuntal or embodied. Players in a team move together (i.e., are connected) in a movement of correspondence with each other, not in a mechanistic repetition. In a team, individuals offer themselves to one another, yet without losing their identities in the composite whole. Like lines in music, whose harmony lies in the alternating tension and resolution, the individuals possess a feel for one another, an “interstitial differentiation,” and are not simply linked by external accretion (Ingold, 2015). This whole is a correspondence, not an assemblage or a fusion. The behavior of a team does not stand over it or lie behind it but emerges from players’ mutual shaping, within a gathering of forces, established through the engagement of individuals that have their own unique skills. Importantly, this understanding of what a sports team is implies a new understanding of how they can be separated, where it is not a matter of cutting an external connection. Something from the history of connection, from the memory together, is lost. Ingold (2015), argues that if you untie a knotted rope, the rope will retain kinks and bends and will want to curl up into similar conformations as before. The memory is suffused into the very material of the rope, in the torsions and flexions of its constituent fibers. If a new knot is tied the rope will retain a memory of its former association.

Other typologies exist for interpersonal linkages (Thompson, 1967; Bell and Kozlowski, 2002), used in organizational management, with recent applications to sport (Reynolds and Salas, 2016). These four types of interdependence are: (i) *pooled interdependence* where each person makes a discrete contribution to the whole; (ii) *sequential interdependence* where a player X must act before player Y can act. We argue that these two types are included in the aggregation type of linkage; (iii) *reciprocal interdependence* requiring player X to act so that player Y can act and player Y’s actions then impact player X’s next action. This type of linkage is, in our view, a type of correspondence linkage. And finally, (iv) *intensive interdependence* in which team members interact simultaneously during task performance. This type of interaction may be related to fusion and aggregation, unless we can trace the contribution of each member to the team and understand how the team influences an element’s behavior. In this case we have correspondence.

In operational terms, there are assembly methods such as measures of heat maps, major ranges. Heat maps provide a clear picture of the distribution of each player on the field. Heat maps highlight with warmer colors the zones where each player has lingered for larger periods of time during the match (Araújo et al., 2015). Another approach to assess the division of labor in team sports is by measuring the area covered by each player. Major ranges imply the calculation of an ellipse centered at each player’s locus and with semi-axes being the standard deviations in the x- and y-directions, respectively (Yue et al., 2008). Through the simple visualization of major ranges it is possible to identify preferred spatial positions, major roles for each player and playing styles (Araújo et al., 2015). A more dynamic view of aggregation can be captured by player-to-locus distance, and Voronoi cells, which contrary to the former two measurements, the distance of each player to a private locus on field, over time, capture the time-evolving nature of their movements’ trajectories. The locus represents the player’s spatial positional reference around which he/she oscillates (McGarry et al., 2014). Individual playing areas attributed to each player on a team, delimited the Voronoi cells of players in team ball sports, and offer a time-evolving analysis of the trajectories of these areas (Fonseca et al., 2013). A Voronoi cell contains all spatial points that are nearer to the player to whom that cell is allocated than to the other players. By measuring the total area of all Voronoi cells from each team, it is possible to obtain a dominant ratio of one team over the other (Fonseca et al., 2012). The view of Latash (2008) of sharing patterns seems to be more related to fusion and captured by the methods described in our discussion of dimensional compression. However one of such methods, cluster phase, can capture simultaneously interpersonal linkages, in a way more related to “correspondence.” Cluster phase measures assess not only synchronies between whole teams, but also between individual players with their team as a function of time, ball possession and field direction (Duarte et al., 2013). In fact Duarte et al. (2013) showed that in player-team synchronization, players tended to be coordinated to different extents under near in-phase modes (near total synchronization) with the team.

### Degeneracy: “To a Good Rider, Right or Left Makes No Difference”

Understanding synergies is far more than identifying and understanding the functional structure of each individual synergy. In addition, we need to understand how one synergy can transform into another at specific moments and/or in specific spatial orientations, how different synergic functions can be incorporated, how distinct synergies can co-exist in the same system elements, and how individual components, constituting a synergy, can be added or withdrawn specific to changing performance circumstances (Turvey, 2007).

Bernstein (1967) emphasized that degrees of freedom are temporarily coordinated together according to circumstances of a performance environment and task requirements. The varying role of synergy degrees of freedom in assembling actions is essential, and is exemplified by the degenerate networks existing at different levels of human movement systems (Seifert et al., 2016). Degeneracy refers to structurally different components (e.g., players in a team) performing a similar, but not necessarily identical, function with respect to context (Edelman and Gally, 2001). In this sense, behavioral adaptability reflects the modification of one individual component in a synergy and/or a whole modification of coordination realized by ‘redundant’ elements (i.e., the presence of isomorphic – same components – and isofunctional components – similar function), or more generally by ‘degenerate’ elements (i.e., the presence of heteromorphic – different elements – variants that are isofunctional; see Mason, 2010). Degeneracy signifies that an individual can vary his/her motor behavior (structurally) without compromising function (Mason, 2010), providing evidence for the adaptive and functional role of coordination pattern variability in order to satisfy changing task constraints (Komar et al., 2014; Seifert et al., 2016). Degeneracy may emerge in a synergy of components that performs a function. It signifies that, regardless of whether some components are able to perform an initial function independently, other components are available for modification (Seifert et al., 2016), supporting interchangeability of different components. Importantly, adaptive team behaviors, where degeneracy is well-exploited, signify that the perception of shared affordances (opportunities for action) is stable when needed, and flexible when needed. Notably, flexibility is not a loss of stability but, conversely, is a sign of adaptability (i.e., a perceptual and motor adaptions to interacting constraints), in order to facilitate (structural or not) changes in coordination patterns and at the same time maintaining functional performance (Seifert et al., 2016).

The functional role of movement variability in sport performance exemplifies how degeneracy emerges at a team level in sport (Davids et al., 2006; Seifert et al., 2016). Performance in a team ball game is sustained by continuous adaptive interactions among players (Araújo et al., 2015). The behavior of such complex systems emerges from the orchestrated activity of many system components (players) that adaptively interact through pairwise local interactions. A common feature of such complex, social networks is that any two nodes or system individuals can become interconnected for action through a path of a few links only (Newman, 2003). Studies of complex networks have revealed that certain forms of network growth produce scale-free networks, that is, the distribution of connections per node in the networks is scale invariant (Barabási and Albert, 1999), as happens with phase transitions and critical points in the dynamics of order parameters. This observation indicates that, degeneracy, as a major synergetic property, might be quantified by different metrics of social networks.

Passos et al. (2011) showed that social networks could be used to analyze the local structure of organization among players, during sub-phases of play in team sports. In these networks, nodes represent players, and links are weighted according to the number of passes or positional changes completed between players. Players with major competitive roles (centrality) may be easily identified through social network analyses, since they display stronger connections with other players. Additionally, different match networks can be compared to extract the tactic behavioral patterns of a team under changing competitive conditions, such as the: (i) in-degree that measures the number of players who pass the ball to a focal player; (ii) out-degree that measures the number of players to which the focal player passes the ball; and (iii), preferential attachments between some team members in certain matches (Duch et al., 2010; Passos et al., 2011; Grund, 2012). However, it is possible to advance the understanding of degeneracy in team sports performance, by using other existing metrics that consider more than the links between a focal node and its neighbors. For example, for understanding the playing style of a sports team, Gyarmati et al. (2014) identified and quantified connection patterns. Fewell et al. (2012), on the other hand, included other metrics such as flow centrality which provides a quantification of individual and team performance regarding a specific task goal such as a shooting attempt at a target (at a basket or at a goal).

Emergent patterns of interaction have been studied using different representations of the interactions between the different individuals. These include hypernetworks, where hyperlinks may connect more than a pair of nodes. This latter approach has been applied to analysis of robotic soccer (Johnson and Iravani, 2007) and has proven particularly powerful.

In summary, networks are a valuable tool to analyze the functional variability during sub-phases of play in team sports, since they facilitate identification of players engaged in more and less frequent interactions within a team, interacting with the ball and the goal/basket/tryline according to competitive events.

## Conclusion

In this overview paper we have discussed the relevance of key concepts from ecological dynamics, a theoretical framework that has provided a rationale for explaining how specific constraints might impact on team synergies formed by players during competitive performance. It has been found that these ecological constraints shape the perception of shared affordances available for players, which underpin the assembly of interpersonal synergies expressed in collective actions within a team. These important group processes support the formation of synergies. Their key properties have begun to be identified including, dimensional compression, reciprocal compensation and degeneracy, guiding the current meaning of operational variables of relevance for PA, such as team center, team dispersion, team synchrony, and team communication. Theoretical and empirical developments in methods of analysis of team coordination and performance can benefit from a powerful theoretical approach that situates and traces relevant team properties as defined by synergies.

For example, actual networks are static: after defining a time interval and collecting all the observable edges during a set period, a network refers to a discrete aggregated view of the whole system. However, the dynamic behaviors of the network are what strongly affect its functionality and efficiency (Moody et al., 2005). Well-connected nodes can quickly become weakly connected (or even disconnected) over time (Hill and Braha, 2010), which is important to consider in future studies because each dynamical system is constrained by universal principles, but at the same time requires its unique suite of analytical and numerical tools to understand its behaviors (Barzel and Barabási, 2013). Moreover, in sport teams, spatial constraints captured in playing areas, rigorously limit, mark, and focus performance behaviors, e.g., a target area, such as a goal, hoop or tryline, has a strong effect on network connectivity patterns. This observation indicates where it is important to develop metrics for analysis and modeling, clarifying where performers attain different impacts, according to their relative positioning, considering key constraints as well as previous performance contributions in a competitive match.

In this paper, we have suggested how concepts like shared affordances, and synergies, framed in an ecological dynamics perspective, present key principles to substantiate the meaning of existing and future operational metrics in PA, and inform about team performance and training development.

## Author Contributions

DA, KD: discussed the structure of the paper; DA: lead the writing of the ms; KD: refined the writing of the paper.

## Conflict of Interest Statement

The authors declare that the research was conducted in the absence of any commercial or financial relationships that could be construed as a potential conflict of interest.
